# Fetal growth outcomes following peri-implantation exposure of Long-Evans rats to noise and ozone differ by sex

**DOI:** 10.1186/s13293-019-0270-6

**Published:** 2019-12-02

**Authors:** Colette N. Miller, Urmila P. Kodavanti, Erica J. Stewart, Mette C. Schladweiler, Judy H. Richards, Samantha J. Snow, Andres R. Henriquez, Wendy M. Oshiro, Aimen K. Farraj, Mehdi S. Hazari, Janice A. Dye

**Affiliations:** 1 Cardiopulmonary Immunotoxicology Branch, Public Health & Integrated Toxicology Division, Center for Public Health & Environmental Assessment U.S. Environmental Protection Agency, Research Triangle Park, NC USA; 2 Oak Ridge Institute of Science and Education, Research Triangle Park, NC USA; 3 ICF International, Inc, Durham, NC USA

**Keywords:** Ozone, Noise, Implantation, Pregnancy, Intrauterine growth restriction, Sex differences

## Abstract

**Background:**

Exposure to air pollution and high levels of noise have both been independently associated with the development of adverse pregnancy outcomes including low birth weight. However, exposure to such environmental stressors rarely occurs in isolation and is often co-localized, especially in large urban areas.

**Methods:**

The purpose of this study was to compare the effects of combined exposure to noise (N) or ozone (O_3_), compared to either exposure alone. Long-Evans dams were exposed to air or 0.4 ppm ozone for 4 h on gestation day (GD) 5 and 6, coinciding with implantation receptivity. A subset of dams from each exposure group was further exposed to intermittent white noise (~ 85 dB) throughout the dark cycle following each inhalation exposure (*n* = 14 − 16/group). Uterine artery ultrasound was performed on GD 15 and 21. Fetal growth characteristics and indicators of placental nutrient status were measured at GD 21.

**Results:**

Exposure to ozone + quiet (O_3_ + Q) conditions reduced uterine arterial resistance at GD 15 compared to air + quiet (A + Q) exposure, with no further reduction by GD 21. By contrast, exposure to air + noise (A + N) significantly increased uterine arterial resistance at both GD 15 and 21. Notably, while peri-implantation exposure to O_3_ + Q conditions reduced male fetal weight at GD 21, this effect was not observed in the air + noise (A + N) or the ozone + noise (O_3_ + N) exposure groups. Fetal weight in female offspring was not reduced by ozone exposure alone (O_3_ + Q), nor was it affected by air + noise (A + N) or by combined ozone + noise (O_3_ + N) exposure.

**Conclusions:**

These data indicate that exposure to ozone and noise differentially impact uterine blood flow, particularly at mid-gestation, with only ozone exposure being associated with sex-dependent fetal growth retardation in male offspring.

## Background

Exposure to environmental pollutants during gestation is associated with the development of adverse pregnancy outcomes. More specifically, preterm birth, preeclampsia, intrauterine growth restriction (IUGR), and low birth weight have all been reported to have a positive relationship with gestational exposure to air pollution [1, 2]. Despite considerable epidemiological investigation, a paucity of causal evidence is available supporting these associations. Taking this into account, we have recently demonstrated that exposure to ozone (an oxidant air pollutant) during implantation receptivity resulted in reduced fetal weight at gestation day (GD) 21 in Long-Evans rats [3, 4]. While dams do not develop preeclampsia following exposure, our findings support the hypothesis that implantation is a critical window in gestation that when disrupted may increase the risk of adverse pregnancy outcomes such as IUGR [5]. We have further shown that ozone exposure appears to alter systemic circulating factors that in turn impair trophoblast viability and invasion in an in vitro model [6]. Such effects may have led to downstream impacts on placentation and vascular remodeling, as we have demonstrated in our ozone-induced rat model of growth restriction.

Importantly, however, exposure to increased concentrations of ozone may not occur in isolation. In the typical urban environment, individuals may be exposed to ozone as a component of complex air pollutant mixtures and also to psychosocial stressors including noise pollution. The near-road environment, for example, is characterized by high levels of both air pollution and noise (upwards of 80 dB) [7]. While ground-level ozone is not described as a near-road pollutant, ozone is a secondary by-product from reactions involving nitrogen oxides and volatile organic compounds that are released from tail pipe emissions, hence, contributing to the complex urban exposome. Similar to air pollution, traffic-related noise pollution has been suggested to be an independent risk factor for the development of both acute and chronic cardiovascular conditions [8, 9]. These relationships are hypothesized to be attributed to activation of the stress response, particularly, stimulation of the hypothalamic-pituitary-adrenal (HPA) axis as suggested by findings in animals exposed to 60–90 dB of varying types of noise including traffic recordings [10–12].

Traffic-related noise has also been associated with adverse pregnancy outcomes including gestational hypertension, congenital malformations, and low birth weight in humans [13, 14]. Such adverse effects on pregnancy have also been reported in animal models. For example, in pregnant mice, exposure to noise reduced placental blood flow, [15], impaired fetal growth [16], and programmed offspring for HPA dysfunction in adulthood [17]. Despite these findings, recent epidemiological reports suggest that air pollutant exposure may act as a confounding factor in the relationship between noise and health outcomes given their colocalization, making it difficult to understand the true influence of gestational noise exposure on fetal health [18, 19]. Improved understanding of the comparative effects of exposure to noise and air pollution alone, or in combination, on gestational outcomes in a controlled setting is currently needed.

The purpose of this study was to determine if exposure to noise pollution during implantation alters fetal weight gain either independently or in interaction with co-exposure to air pollution. To assess the potential for an interaction with an air pollutant, we utilized our previously established model of ozone-induced growth restriction [3, 4]. In our previous work, we determined that both male and female fetuses from dams exposed to 0.8 ppm ozone (4 h) on GD 5 and 6 had reduced fetal weight. However, when dams were exposed to 0.4 ppm ozone (4 h) on GD 5 and 6, the impaired fetal weight gain was only observed in male fetuses [3]. Thus, herein, to better investigate the potential for an interaction between co-exposure to noise and air pollution, dams were exposed during implantation receptivity to the low ozone concentration (0.4 ppm) plus intermittent white noise during their dark cycle following each ozone exposure on GD 5 and 6. Changes in uterine vascular resistance were assessed using Doppler ultrasonography at GD 15 and 21 and fetal growth endpoints and placental metabolic status were assessed at GD 21. Given the previous findings linking exposure to noise and reduced fetal growth [16], we hypothesized that acute exposure to noise alone during implantation would likewise impair fetal growth in rats. Moreover, we hypothesized that combined exposure to noise and 0.4 ppm ozone would have an interactive effect on the risk of growth restriction, further reducing fetal weight in males and leading to significant reductions in females relative to the effects of 0.4 ppm ozone-only exposures.

## Methods

### Animals and ozone exposure

Timed-pregnant Long-Evans dams (11-week old, 200–220 g) arrived at GD 1 (plug-positive day) from the local Charles River Laboratory facility (Raleigh, NC). Dams were single-housed in plexiglass cages and were fed a phytoestrogen-free, American Institute of Nutrition growth and lactation diet (D15092401; Research Diets), provided ad libitum. Body weight was monitored daily throughout the study. Daily food intake (g) was calculated by assessment of food weight, subtracting each day’s food weight from the day previously. The Institutional Animal Care and Use Committee of the U.S. Environmental Protection Agency’s National Health and Environmental Effects Research Laboratory approved of all experiments prior to initiation of the study.

Upon arrival, dams were randomly assigned into one of four exposure groups (*n* = 16/group): filtered air + quiet (A + Q), filtered air + intermittent noise (A + N), 0.4 ppm ozone + quiet (O_3_ + Q), or 0.4 ppm ozone + intermittent noise (O_3_ + N). The concentration of ozone used in the current study is based on a four-to-five fold lung exposure dosimetry difference between resting male rats and intermittently exercising humans, which corresponds to a 0.08–0.10 ppm human exposure concentration [20]. All air or ozone (0.4 ppm) exposures were for 4 h (0700–1100 h) on GD 5 and 6. Ozone was generated using a silent arc discharge generator (OREC^TM^) and delivered to temperature- and humidity-controlled Rochester-style “Hinners” exposure chambers by a mass flow controller. Ozone concentrations were monitored continuously throughout the exposure by an ozone analyzer (API Model 400; Teledyne Instruments, City of Industry, CA).

### Intermittent noise exposure

The noise exposure was produced by intermittent white noise generated at 85 dB by a system developed in-house. The system included a series of speakers (#TS-A1676R, 32 Hz–40 kHz [− 20 dB]; Crutchfield Corporation, Charlottesville, VA), amplifiers (Amp100; AudioSource, Portland, OR), and a noise generator (Type 1405; Bruel & Kjaer, Naerum, Denmark) connected remotely to a relay switch that opened and closed noise output via a programmable outlet strip (#94450-10; Cole Parmer, Vernon Hills, IL). Each speaker (6-in diameter) was mounted 1.25 in above the middle of each cage, and the programmable timer was used to gate power to the system allowing variable noise durations ranging from 5 to 25 min, with 50 to 180 min between each exposure. Rats were exposed to the intermittent white noise following the air or ozone exposure during the dark cycle on GD 5 and after the second inhalation exposure on GD 6. In order to not disrupt sleep patterns [21], exposure to noise occurred solely during the dark cycle. The initial noise exposure began when the lights were turned off at 1800 h and the final exposure ending shortly before the light cycle at 0600 h. In all, the cumulative noise exposure was 85 min per day.

**Fig. 1:**
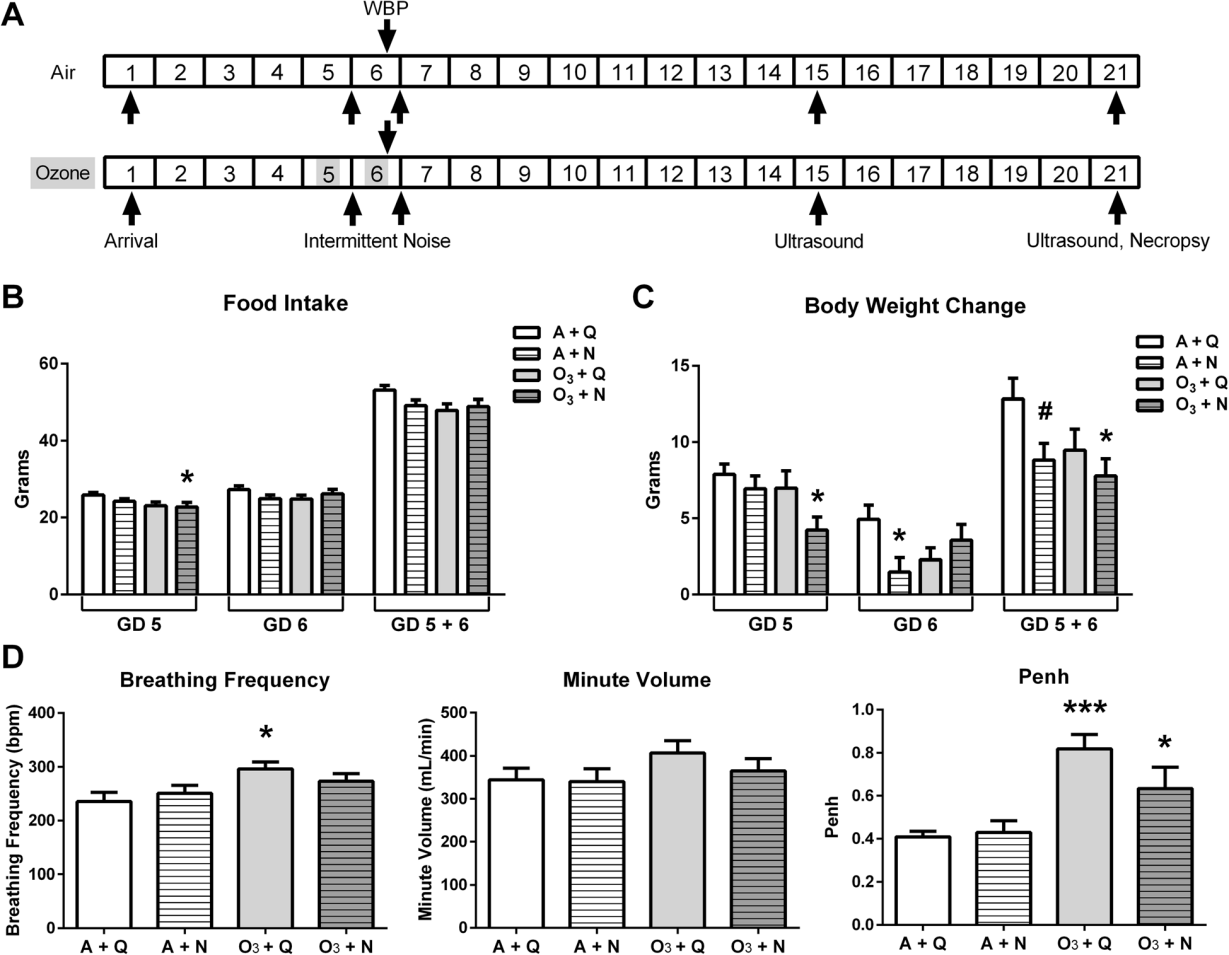
Acute maternal food intake, body weight gain and whole-body plethysmography endpoints at gestation day 5 and 6. The design of the study (**a**). Food intake (**b**) and body weight change (**c**) were assessed during the exposure period on gestation days 5 and 6 and are presented as both the 24-h difference and 48-h difference (*n* = 14–16/group). Breathing frequency, minute volume, and Penh (**d**) were assessed using whole body plethysmography following the second ozone exposure on GD 6 (30-min–1-h post-exposure; *n* = 8–10/group). Data are presented as mean ± standard error and were analyzed by Dunnett’s post-test within a two-way ANOVA. ^#^*p* < 0.10, *p < 0.05, ***p < 0.001 *vs.* A + Q group. Abbreviations: whole body plethysmography (WBP), gestation day (GD), air + quiet (A + Q), air + noise (A + N), ozone + quiet (O_3_ + Q), ozone + noise (O_3_ + N)

Noise levels were tested for each cage prior to the start of the experiment. We have previously determined that noise levels in the four corners of the cage vary from 80 to 85 dBA, while levels at the top middle of the cage (nearest the speaker) will vary from 90 to 95 dBA. Thus, depending on the movement and location of the rat during the noise exposure, rats will experience a range of white noise intensity of ~ 80 to 95 dB while the speakers are active. These levels are similar to what is experienced from a food blender. Furthermore, noise ranging 80–95 dB is not known to cause hearing damage in rats [22]. To maintain consistent handling of dams exposed to noise vs. “quiet,” the dams of the quiet groups were similarly transported to a new room and single-housed during their corresponding period of testing. On GD 7, all dams were returned to their original home-cage room. A visual representation of the study design is presented in Fig. 1a.

### Whole body plethysmography

To assess the extent of ozone-induced acute ventilatory dysfunction, immediately after termination of the second day of exposure (GD 6), a subset of dams was transported to an adjacent room and placed in unrestricted whole-body plethysmographs. Rats were allowed 3 min to acclimate and then data were acquired for 5 min. Due to unforeseen circumstances (adverse weather conditions), group sizes were *n* = 10 for the A + Q group and *n* = 8 for all other groups. Breathing frequency, an estimated minute volume, and Penh, an index of airflow limitation (Miller, Dye [23], Hamelmann, Schwarze [24]), were determined using emka iox2 software (emka TECHNOLOGIES, Falls Church, VA).

### Uterine artery Doppler ultrasound

As previously described (3), resistance in the uterine artery was measured using pulsed-wave Doppler ultrasonography (Vevo® 2100 Imaging System; VisualSonics, Inc., Toronto, Ontario). Due to the aforementioned unexpected weather event the group sizes were *n* = 12 for the A + Q and A + N groups, *n* = 13 in the O_3_ + Q and O_3_ + N groups. Induction of anesthesia was achieved with 3% isoflurane and maintained at ~ 1.5% to ensure a stable heart rate at 400 ± 25 beats per minute. Abdominal fur was shaved using clippers, followed by application of a depilatory agent. The uterine artery was identified using Color Doppler imaging and measurements of uterine arterial velocity were taken at the location of the ascending left uterine artery cranial to the “U-turn” to ensure consistency. Pulse-waved Doppler was obtained using a 12.5 MHz MS201 transducer (VisualSonics, Inc., Toronto, Ontario). Six pulsatile blood flow waveforms were analyzed in a blinded manner to calculate indicators of arterial resistance including peak systolic volume (PSV), end diastolic volume (EDV), and resistance index (RI) (Vevo Lab software, v.1.7, VisualSonics, Inc., Toronto, Ontario). The RI was calculated using the formula: (PSV—EDV)/PSV.

### Necropsy

On GD 21, dams and fetuses were euthanized by a lethal intraperitoneal injection of pentobarbital (> 400 mg/kg) following a 5–6 h fast. Whole blood was collected via the abdominal aorta into serum separator tubes and centrifuged for 10 min at 2465×*g* and 4 °C; serum was stored at – 80 °C until use. The gravid uterus was weighed, assessed for implantation sites, and dissected to obtain fetal and placental tissue. For each fetus, body weight, sex, crown-to-rump length, and corresponding placental weight were determined. Placental efficiency was calculated by fetal weight/placental weight in mg. Lastly, the right and left ovaries were dissected, placed in saline, and the total number of corpora lutea were enumerated. Based on a priori determined inclusion criteria, dams were excluded from study if their total litter size was *n* ≤ 3 or if pre- or post-implantation loss was *n* ≥ 7. Such findings are consistent with underlying pregnancy conditions unrelated to ozone or noise exposure. Final group numbers are presented in Table 1. Notably, only an *n* = 1 to 2 were removed from three groups, and no dams were excluded from the O_3_ + N group.

**Table 1:** Litter characteristics at gestation day 21

Characteristics	ANOVA	A + Q	A + N	O_3_ + Q	O_3_ + N
Extrauterine weight gain (g)	–	150 ± 5.27	141 ± 4.68	149 ± 4.36	150 ± 4.76
Total gestational food intake (g)	–	493 ± 12.6	473 ± 15.0	478 ± 14.3	496 ± 19.6
Total corpora lutea	–	14.3 ± 0.37	14.9 ± 0.45	14.0 ± 0.49	15.1 ± 0.63
Implantation sites per litter	–	12.7 ± 0.41	12.9 ± 0.42	13.1 ± 0.55	13.8 ± 0.56
Resorptions per litter	–	0.71 ± 0.22	0.73 ± 0.23	0.73 ± 0.25	0.94 ± 0.19
Full term fetuses	–	12.0 ± 0.38	12.1 ± 0.49	12.3 ± 0.47	12.9 ± 0.50
Percent males per litter	–	53.1 ± 2.49	51.1 ± 3.31	45.2 ± 4.40	49.5 ± 3.19
Total litters ^excluding a,b,c^		14^a,b^	15^c^	15^b^	16

Data are presented as mean ± standard error and analyzed by Dunnett’s post-test within a two-way ANOVA. The second column details if a significant effect or interaction determined by two-way ANOVA for each endpoint was found; however, no significant effect or interaction was measured

*A + Q* air + quiet, *A + N* air + noise, *O*_*3*_ *+ Q* ozone + quiet, *O*_*3*_ *+ N* ozone + noise

Dams were removed from study if ^a^post-implantation loss was *n* ≥ 7, ^b^pre-implantation loss (corpora lutea–implantation sites) was *n* ≥ 7, or ^c^total litter size was *n* ≤ 3, effects suggestive of an underlying pregnancy complication unrelated to exposure

### Serum metabolic and hormone assessments

Using serum collected at necropsy on GD 21, the following commercial assays were adapted for use on the Konelab Arena 30 system (Thermo LabSystems, Espoo, Finland): free fatty acids (Cell Biolabs, Inc., San Diego, CA); high density lipoprotein (HDL) and low density lipoprotein (LDL) cholesterol (Sekisui Diagnostics, Lexington, MA); glucose, total cholesterol, and triglycerides (TECO Diagnostics, Anaheim, CA). 17β-estradiol and progesterone were assessed using ELISA kits by Enzo Life Sciences (Farmingdale, NY) using manufacturer instructions following ether precipitation. The ELISAs were read using a SpectraMax i3x (Molecular Devices, San Jose, CA.

### Placental metabolic assessment

Subsets of placental samples were selected across litters with matching male and female pairs (*n* = 12/group/litter/sex). Using tissue from the fetal side of the placenta, samples (~ 75 mg) were homogenized in ice-cold RIPA buffer containing protease inhibitors. The homogenate was centrifuged at 10,000×*g* for 10 min at 4 °C, and the supernatant was collected, taking care to include the lipid layer, and frozen at − 80 °C until further processing and use. The following commercial kits were adapted for use on the Konelab Arena 30 system (Thermo LabSystems, Espoo, Finland): free fatty acids (Cell Biolabs, Inc., San Diego, CA); glutathione peroxidase activity (GPx; adapted from Jaskot, Charlet [25]), superoxide dismutase (SOD) activity, and total antioxidant status (TAS; Randox Laboratories, Ltd, United Kingdom); total protein (Coomassie Plus Protein Assay Kit; Pierce Scientific; Rockford, IL); and glucose, total cholesterol, and triglycerides (TECO Diagnostics, Anaheim, CA). Because the protein concentration of the placental tissue was impacted by exposure, all placental data were adjusted by the weight of the homogenized sample.

### Fetal body composition assessment

Fetal fat and lean mass were measured by magnetic resonance using the Bruker ‘minispec’ LF90 II analyzer (Bruker Optics, Inc., Billerica, MA). Due to the minimum weight requirement for analysis (5 g), groups of *n* = 3 fetuses/sex/dam were randomly selected and pooled for measurement. If there was an insufficient number of fetuses available to be pooled, no measurements were obtained for that litter’s sex. Therefore, final group sizes were *n* = 14 (A + Q), *n* = 15 (A + N), *n* = 13 (O_3_ + Q), and *n* = 16 (O_3_ + N). Fat-to-lean mass ratios were calculated by dividing the lean body mass (grams) by fat mass (grams).

### Statistics

Group sizes were based on our previous work demonstrating a significant effect of 0.4 ppm ozone exposure on fetal weight in male offspring using *n* = 9 dams/exposure group [3]. Herein, we increased our group sizes to an *n* = 16 to account for the difference in study design. The effects of ozone and noise exposure were tested by two-way ANOVA and post-testing was performed by Dunnett’s multiple comparison. If data were not normally distributed (e.g., fetal weight, placental weight, and placental efficiency), ANOVA was not performed, and between group differences were tested by Dunn’s multiple comparisons post-test. Effect size, which measures the magnitude of the difference between two groups, was calculated for selected endpoints, namely Penh and fetal body weight, using Hedges’s *g* corrected for unequal sample size. For these parameters, the Hedges’s *g* is presented along with the 95% confidence interval (CI). Effect size can be used as a simple guideline to understand the magnitude of the exposure-related outcome and can be stratified as relatively small (0.2–0.5), medium (0.5–0.8), and large (≥ 0.8) [26]. Lastly, one-tailed, chi-squared analysis was used to assess relationships between acute Penh changes post-ozone exposure in dams on GD 6, with fetal weight on GD 21. Graphical data are presented as the mean and standard error, and the critical alpha level was set at *p* < 0.05. Statistics were performed, and graphs were prepared using GraphPad Prism (v.6.07, La Jolla, CA).

## Results

### Acute effects of exposure on maternal food intake and body weight

Acute exposure to 0.4 ppm ozone for 4 h decreased daily food intake in dams (Fig. 1b). Based on two-way ANOVA factorial analysis, there was an independent (main) effect of ozone exposure after GD 5 (*p* < 0.05; f[1, 56] = 5.56). Compared with the A + Q group, food intake was significantly reduced only in the O_3_ + N at GD 5 (Fig. 1b; *p* < 0.05). By the second day of exposure, no differences in food intake were observed. Both ozone and noise exposure had significant independent (main) effects on body weight gain following the first day of exposure on GD 5 (*p* < 0.05; f[1, 54] = 4.08 and *p* < 0.05; f[1, 54] = 4.28, respectively). Accordingly, compared with the A + Q group, maternal weight gain was reduced in the O_3_ + N group following the first day of exposure (Fig. 1c; *p* < 0.05). After the second day of exposure, compared with the A + Q control group, maternal weight gain was reduced only in the A + N group (Fig. 1c; *p* < 0.05). Thus, for the combined exposure period only noise resulted in a significant independent (main) effect on body weight gain (*p* < 0.05; f[1, 54] = 5.22). The O_3_ + N group had reduced weight gain for the combined GD 5–6 period (Fig. 1c; *p* < 0.05).

### Acute effects of exposure on maternal ventilation

Immediately following the second inhalation exposure on GD 6, dams underwent whole-body plethysmography. The two-way ANOVA showed a significant independent (main) effect of ozone to increase breathing frequency (*p* < 0.05; f[1, 30] = 7.22) and Penh (*p* < 0.0001; f[1, 30] = 23.2). More specifically, increases in breathing frequency (*p* < 0.05) and Penh (*p* < 0.001) were observed in the O_3_ + Q group compared with the A + Q control group (Fig. 1c). Of note, dams exposed to O_3_ + N did not exhibit increased breathing frequency. Penh, while increased in the O_3_ + N group compared with the A + Q controls (Fig. 1c; *p* < 0.01), did not appear to be as consistently affected as the O_3_ + Q group. We therefore used the Hedges’s *g* calculation to quantify the effect size of the Penh increases. Seemingly, concurrent exposure to both O_3_ + N reduced the effect size of the Penh response (*g*_Hedges_ = 1.15 [95% CI 0.15, 2.16]) relative to that observed for the O_3_ + Q group (*g*_Hedges_ = 2.95 [95% CI 1.61, 4.29]). Put another way, there was more variability in Penh in dams exposed to O_3_ + N compared with ozone alone, thereby reducing the overall effect of ozone exposure on Penh.

### Changes in uterine arterial resistance in mid-to-late pregnancy

**Fig. 2:**
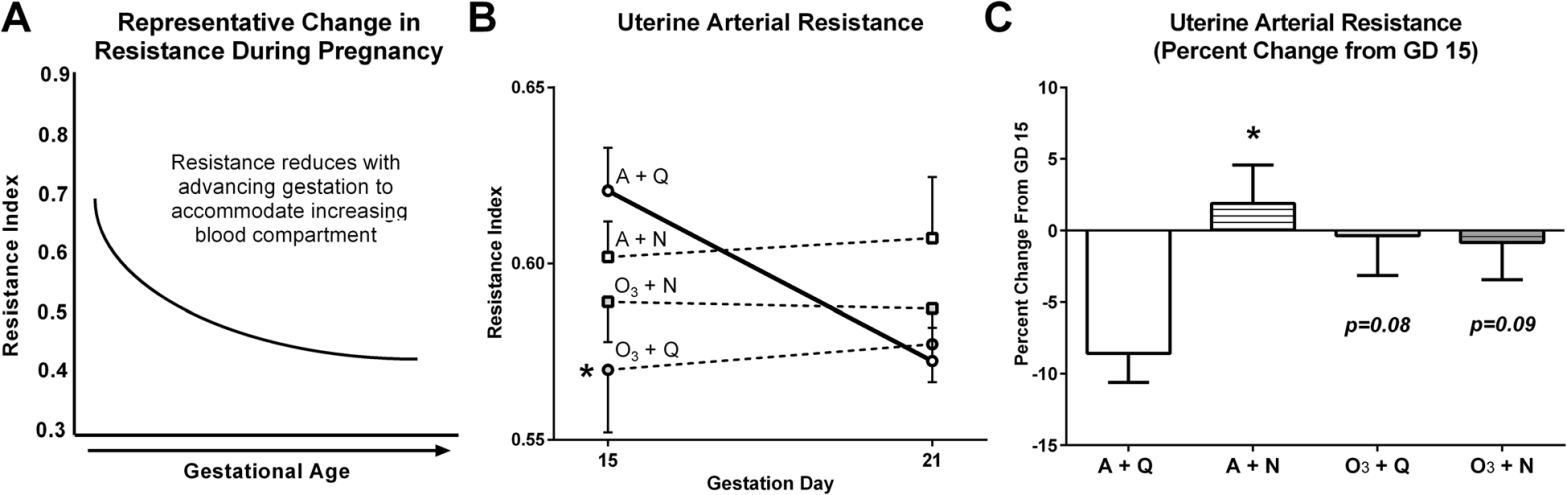
Uterine arterial resistance at gestation day 15 and 21. Uterine arterial resistance was measured on gestation days 15 and 21. A representative pattern of the resistance change that occurs in the uterine artery in a normal pregnancy (**a**). The calculated resistance index (**b**) is shown for both days of measurement, and the percent change from GD 15 to 21 is shown in panel C. Data are shown as mean ± standard error and were analyzed by Dunnett’s post-test within a two-way ANOVA (*n* = 12–13/group). *p < 0.05 *vs.* A + Q. Specific *p* values between groups are presented for clarity. Abbreviations: gestation day (GD), air + quiet (A + Q), air + noise (A + N), ozone + quiet (O_3_ + Q), ozone + noise (O_3_ + N), resistance index (RI)

In a healthy pregnancy, uterine arterial resistance decreases with advancing gestation (Fig. 2a). We therefore assessed uterine arterial resistance by Doppler ultrasonography on GD 15 and 21. Peri-implantation ozone exposure resulted in a significant independent (main) effect to decrease resistance at GD 15 (*p* < 0.05; f[1, 46] = 5.63). Specifically, RI was reduced in dams from the O_3_ + Q group compared with A + Q controls (Fig. 2b; *p* < 0.05). Exposure to O_3_ + N failed to induce the same effect size in uterine arterial resistance as the O_3_ + Q group at GD 15 (RI *g*_Hedges_ = − 0.771 [− 1.59, − 0.04] vs. − 0.934 [− 1.76, − 0.11], respectively), and exposure groups were not significantly different from the A + Q controls. No group differences were found by GD 21.

We next calculated the percent change of RI between GD 15 and 21. In the healthy A + Q group, RI was reduced by ~ 8.6% to better accommodate the expanded blood compartment associated with advanced pregnancy. By contrast, two-way ANOVA indicated a significant interaction between ozone and noise in the percent reduction in uterine artery RI (*p* < 0.05; f[1, 46] = 4.59). Specifically, the RI of the A + N group increased slightly compared with A + Q controls (Fig. 2c; *p* < 0.05), while the O_3_ + Q and O_3_ + N groups were essentially unchanged (Fig. 2c). Hence, on average, dams exposed to ozone, noise, or a combination of both during implantation receptivity failed to induce the same extent of reduction in resistance through the uterine artery in the second half of gestation.

### Pregnancy-related endpoints at GD 21

At necropsy on GD 21, no differences were observed in extrauterine weight gain or total gestational food intake (Table 1). No differences were observed for pregnancy endpoints including corpora lutea number, implantation sites, resorptions, and the number of fetuses and percent males per litter (Table 1). Together, these data suggest that exposure to 0.4 ppm ozone, intermittent noise, or a combination of both had no overt impact on uterine endpoints and sex distribution of the fetuses by GD 21.

### Changes in serum metabolic parameters and hormones at GD 21

Serum was obtained at necropsy on GD 21 to determine if exposure to ozone and noise during implantation receptivity affected circulating metabolic endpoints in the dam, some of which (glucose and free fatty acids) we have previously reported to be changed following exposure to 0.4 ppm ozone [3]. Two-way ANOVA analysis showed a significant independent (main) effect of noise exposure to decrease total cholesterol levels (*p* < 0.01; f[1, 56] = 7.24) and a significant main effect of ozone to increase serum LDL cholesterol (*p* < 0.05; f[1, 56] = 4.72); however, there were no differences between A + Q controls and the other exposure groups (Table 2). Furthermore, there were no differences in serum glucose, HDL cholesterol, or triglyceride concentrations and no measurable alterations in serum 17β-estradiol or progesterone at GD 21 (Table 2).

**Table 2:** Maternal metabolic panel and hormones at gestation day 21

Serum parameter	ANOVA	A + Q	A + N	O_3_ + Q	O_3_ + N
Glucose (mg/dL)	–	98.3 ± 2.64	97.7 ± 2.61	95.9 ± 2.33	94.4 ± 1.67
Total cholesterol (mg/dL)	Noise	109 ± 4.46	104 ± 6.13	119 ± 5.66	94.1 ± 5.80
HDL cholesterol (mg/dL)	–	38.8 ± 0.90	37.2 ± 0.69	40.2 ± 0.89	39.8 ± 1.54
LDL cholesterol (mg/dL)	Ozone	12.8 ± 0.60	11.3 ± 0.26	13.9 ± 0.89	13.1 ± 0.76
Triglycerides (mg/dL)	–	181 ± 21.0	211 ± 14.9	210 ± 17.1	206 ± 23.2
17β-estradiol (pg/mL)	–	42.1 ± 5.32	34.5 ± 2.65	36.9 ± 5.29	78.8 ± 24.2
Progesterone (ng/mL)	–	19.4 ± 1.85	18.5 ± 0.99	17.5 ± 1.41	19.8 ± 1.93

Data are presented as mean ± standard error and analyzed by Dunnett’s post-test within a two-way ANOVA (*n* = 14–16/group). A significant (main) effect or interaction determined by two-way ANOVA for each endpoint is detailed in the second column

*A + Q* air + quiet, *A + N* air + noise, *O*_*3*_ *+ Q* ozone + quiet, *O*_*3*_ *+ N* ozone + noise, *HDL* high density lipoprotein, *LDL* low density lipoprotein

### Changes in fetal growth measurements at GD 21

**Fig. 3:**
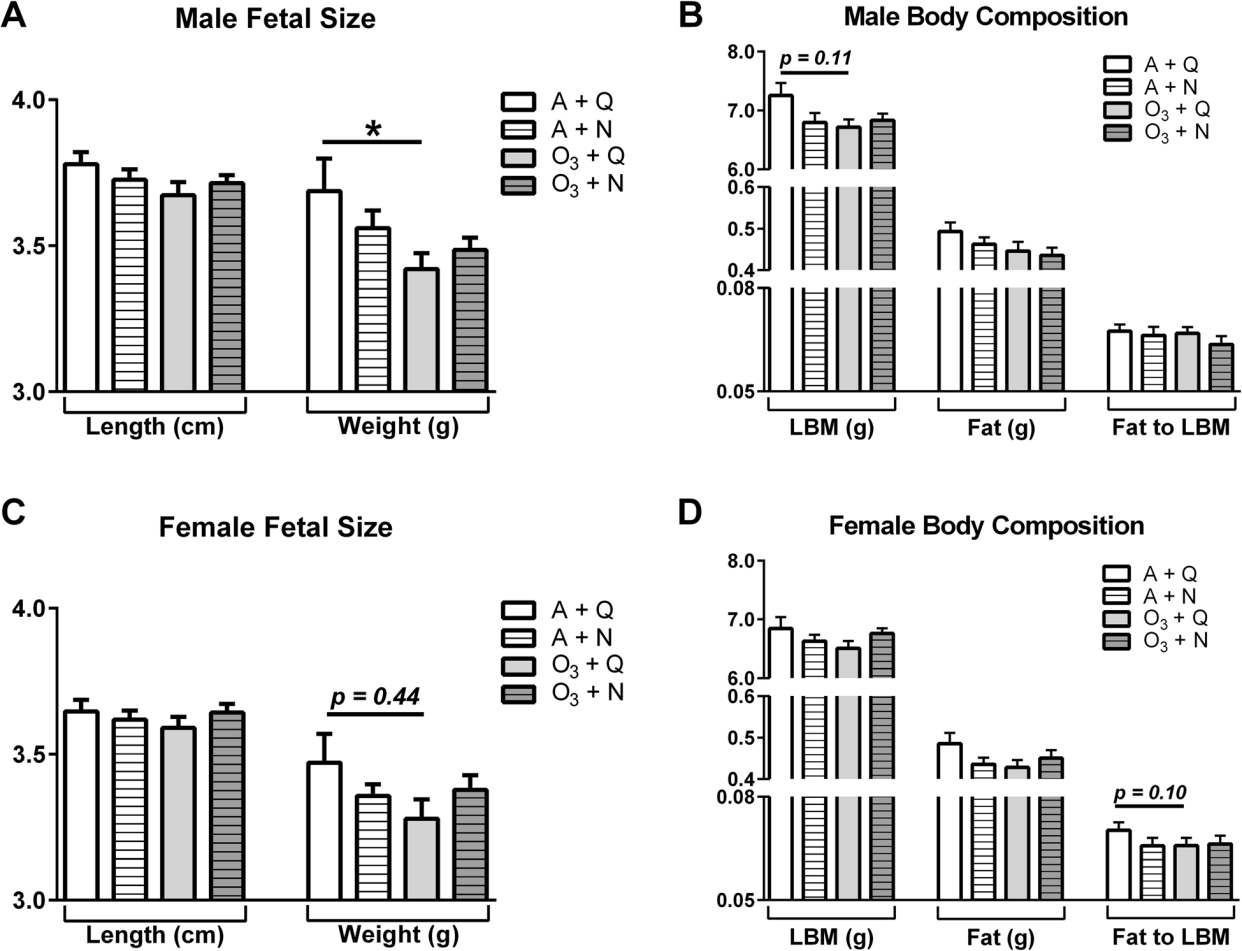
Measurements of fetal growth at gestation day 21. Measurements of fetal length and weight at gestation day 21 are presented for males (**a**) and females in (**c**). Body composition was measured by magnetic resonance in randomly selected, pooled subsets of fetuses (*n* = 3/sex/dam) for males (**b**) and females (**d**). Data are presented as mean ± standard error and were analyzed by Dunn’s post-test for fetal weight, lean body mass, and fat. Fetal length and Fat to LBM ratios were analyzed by Dunnett’s post-test within a two-way ANOVA for all other endpoints. *p < 0.05 *vs.* A + Q. Specific *p* values between groups are presented for clarity. Abbreviations: air + quiet (A + Q), air + noise (A + N), ozone + quiet (O_3_ + Q), ozone + noise (O_3_ + N), lean body mass (LBM)

Like our previous observations in male fetuses [3], exposure to 0.4 ppm ozone during implantation receptivity had no effect on fetal length; however, fetal weight was significantly reduced in the O_3_ + Q group relative to A + Q controls (Fig. 3a; *p* < 0.05). Intriguingly, the effect size of the exposure on fetal weight in the males from the O_3_ + N (*g*_Hedges_ = − 0.65 [− 1.38, 0.09]) group was not as robust as the O_3_ + Q group (*g*_Hedges_ = − 0.81 [− 1.57, − 0.06]), with the latter having the largest absolute value of Hedges’s *g* and indicating a stronger effect size. Hence, there was no difference in fetal weight between the O_3_ + N group and A + Q controls, thereby suggesting that exposure to both ozone and noise exposure fails to impart interactive effects on fetal weight. Additionally, there were no impacts of noise or ozone exposure on fetal body composition including lean body mass, fat mass, or the fat-to-lean mass ratio in males (Fig. 3b). However, lean body mass of the O_3_ + Q group compared with A + Q was trending downward (Fig. 3b; *p* = 0.11).

In female fetuses, the two-way ANOVA failed to find significant effects of either ozone or noise on body length or body weight at GD 21. Thus, unlike the males, female fetal weight at GD 21 from the O_3_ + Q group was not lower than the A + Q controls (Fig. 3c; *p* = 0.44, *g*_Hedges_ = − 0.60 [− 1.35, 0.14]). Lastly, neither ozone nor noise impacted fetal body composition in females (Fig. 3d). Together, the data failed to show an effect of exposure to noise during implantation receptivity on fetal weight.

### Changes in placental metabolic status at GD 21

There were no effects of ozone or noise exposure on placental weight (Table 3). We next calculated placental efficiency (fetal weight in mg/placental weight in mg) as a proxy measurement to assess the placenta’s capacity to transfer nutrients to the fetus. Placental efficiency is often decreased in complicated pregnancies such as intrauterine growth restriction (IUGR) [27], suggesting it may contribute to reduced fetal size. In other words, a reduced fetal to placental weight ratio suggests that the placenta is relatively larger than it should be and less efficient at transferring nutrients to the fetus. Our results showed that noise exposure did not influence placental efficiency in either sex (Table 3). However in both male and female fetuses, the two-way ANOVA showed a significant independent effect of ozone to reduce placental efficiency (*p* = 0.01; f[1, 56] = 6.50 and *p* < 0.05; f[1, 56] = 4.17, respectively), with no specific between-group differences (Table 3).

**Table 3:** Placental endpoints at gestation day 21

	Placental endpoint	ANOVA	A + Q	A + N	O_3_ + Q	O_3_ + N
Males	Placental weight (mg)	–	0.46 ± 0.01	0.43 ± 0.01	0.46 ± 0.02	0.45 ± 0.01
	Placental efficiency	Ozone	8.16 ± 0.29	8.28 ± 0.19	7.52 ± 0.26	7.73 ± 0.17
	Glucose (ng/mg)	–	83.4 ± 4.20	81.8 ± 3.88	88.6 ± 2.72	85.5 ± 3.71
	Protein (μg/mg)	Interaction	72.6 ± 1.83	69.3 ± 2.53	65.8 ± 2.56	72.9 ± 2.02
	Cholesterol (ng/mg)	–	13.6 ± 0.38	12.7 ± 0.43	13.1 ± 0.48	12.6 ± 0.44
	Triglycerides (ng/mg)	–	11.8 ± 0.31	11.6 ± 0.42	12.3 ± 0.58	11.4 ± 0.44
	GPx activity (mIU/mg)	–	7.46 ± 0.34	6.73 ± 0.31	7.13 ± 0.33	7.29 ± 0.32
	SOD activity (mU/mg)	Ozone	6.45 ± 0.32	6.27 ± 0.28	5.91 ± 0.18	5.52 ± 0.26*
	TAS (nmol/mg)	–	12.7 ± 0.49	12.4 ± 0.42	13.1 ± 0.43	12.9 ± 0.55
Females	Placental weight (mg)	–	0.43 ± 0.01	0.43 ± 0.01	0.43 ± 0.01	0.45 ± 0.01
	Placental efficiency	Ozone	8.12 ± 0.25	7.87 ± 0.19	7.65 ± 0.13	7.59 ± 0.17
	Glucose (ng/mg)	Interaction	81.0 ± 4.25	66.1 ± 2.46*	82.2 ± 3.74	86.6 ± 5.38
	Protein (μg/mg)	–	70.7 ± 2.35	64.2 ± 1.42	68.3 ± 2.18	70.8 ± 2.87
	Cholesterol (ng/mg)	–	12.3 ± 0.49	11.7 ± 0.35	12.3 ± 0.41	13.3 ± 0.45
	Triglycerides (ng/mg)	–	12.0 ± 0.56	12.7 ± 0.72	12.2 ± 0.53	12.7 ± 0.57
	GPx activity (mIU/mg)	–	7.00 ± 0.36	6.03 ± 0.25	6.51 ± 0.26	6.71 ± 0.34
	SOD activity (mU/mg)	Noise	5.19 ± 0.24	4.54 ± 0.22	5.33 ± 0.20	5.02 ± 0.27
	TAS (nmol/mg)	Ozone	12.3 ± 0.50	10.8 ± 0.29	12.6 ± 0.39	12.6 ± 0.61

Data are presented as mean ± standard error (*n* = 12/group/sex). Male placental efficiency (fetal weight/placental weight in mg) were analyzed by Dunn’s post-test. All other endpoints were normalized to sample weight and analyzed by Dunnett’s post-test within a two-way ANOVA. A significant (main) effect or interaction determined by two-way ANOVA for each endpoint is detailed in the third column. *p < 0.05 vs. A + Q group

*A + Q* air + quiet, *A + N* air + noise, *O*_*3*_ *+ Q* ozone + quiet, *O*_*3*_ *+ N* ozone + noise, *GPx* glutathione peroxidase, *SOD* superoxide dismutase, *TAS* total antioxidant status

In addition to potential disruptions in placental efficiency, lipid homeostasis may be perturbed in placentas from pregnancies complicated by IUGR [28]. To better assess for comparable changes, we evaluated metabolic parameters in placental tissues. In the male placentas, two-way ANOVA showed a significant interaction between peri-implantation ozone exposure and intermittent noise on protein concentration (*p* < 0.05; f[1, 44] = 5.39). Furthermore, a significant independent effect of ozone to reduce placental SOD activity was measured (*p* < 0.05; f[1, 44] = 6.00). Specifically, male placentas from the O_3_ + N group had reduced SOD activity compared with A + Q controls (*p* < 0.05, Table 3). There were no other differences in placental cholesterol, glucose, triglycerides, or in the antioxidant measurements of GPx and TAS in male placentas.

In female placentas, two-way ANOVA showed a significant interaction between ozone and noise on placental glucose (*p* < 0.05; f[1, 44] = 5.56). Specifically, glucose concentrations in female placentas from the A + N group compared with A + Q controls were reduced (*p* < 0.05). The two-way ANOVA also showed a significant independent effect of ozone to increase placental TAS (*p* < 0.05; f[1, 44] = 5.92) and an independent effect of noise to reduce SOD activity (*p* < 0.05; f[1, 44] = 4.16), without any between group-differences. Lastly, there were no differences in placental cholesterol, protein, triglycerides, or GPx in the female placentas. Hence, neither changes in placental efficiency nor metabolic status explained any relationship, or lack thereof, between exposure to ozone and/or noise and fetal weight.

### Relationships between ozone-induced ventilatory dysfunction and pregnancy outcomes

Surprisingly, the size of the effect of ozone on the weight of both male and female fetuses was reduced if the dams were also exposed to noise during implantation receptivity. Contrary to our initial hypothesis, male and female fetal weights were not reduced in dams exposed to O_3_ + N during implantation receptivity. Because of this result, we sought to further investigate if there was a potential relationship between the degree of ventilatory dysfunction dams experienced acutely after the second ozone exposure (GD 6) with the likelihood of dams’ litters having fetal weight below the median for the A + Q control group. Because Penh is used as an index of airflow limitation [24] and we have shown it to be correlated with pulmonary injury in ozone-exposed rats [23], we utilized it herein as a proxy for the extent of respiratory distress in the individual dams. Then using chi squared analysis, we evaluated whether dams exhibiting a greater degree of distress (Penh > 0.75) were also at increased risk of having small fetuses (i.e., litter fetal weight below the median for the A + Q control group for each sex).

**Fig. 4:**
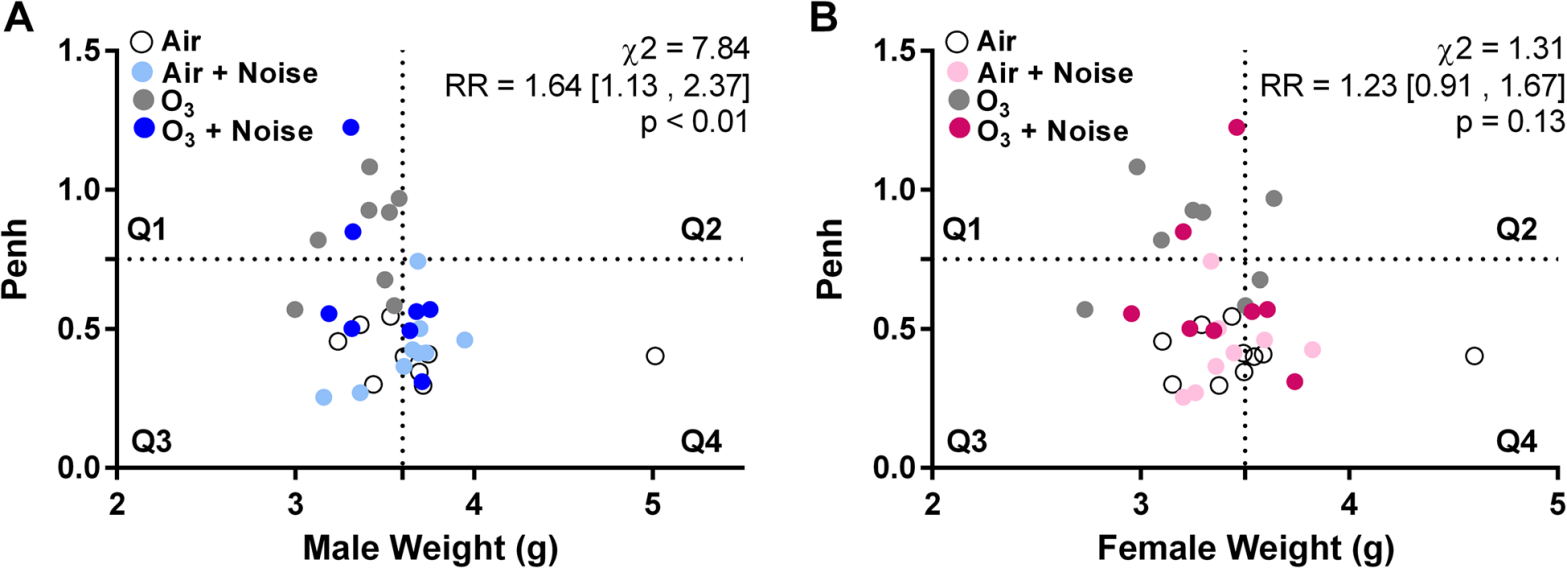
Relationship between acute ventilatory distress on gestation day 6 and fetal weight at gestation day 21. Chi-squared (*Χ*^2^) analysis was used to test relationships between Penh, measured by whole body plethysmography following the ozone exposure on gestation day 6, and fetal weight in males (**a**) and females (**b**) measured on gestation day 21. Group criteria was set based off a 0.75 threshold for Penh and median birth weight from the air + quiet, control group. The strength of the association was tested by relative risk (RR) and is presented alongside the 95% confidence interval

As depicted in Fig. 4, regardless of exposure group, the dams exhibiting Penh increases > 0.75 had male offspring with reduced fetal weight (Fig. 4a; quartile 1). This included O_3_ + Q (5 of 8) and O_3_ + N (2 of 8) dams. Using chi-squared analysis, we observed that dams with an elevated Penh (> 0.75) had male offspring with reduced fetal weights (*Χ*^2^ = 7.84, *p* < 0.01). These same associations were not significant in the female offspring (Fig. 4b).

## Discussion

A growing body of epidemiological evidence suggests that maternal and fetal health may be adversely affected by exposure to air pollution [1] and other environmental stressors such as traffic-related noise [14]. While both ozone and noise are independently associated with the development of adverse pregnancy outcomes, exposure to these types of environmental hazards rarely occur separately [7]. Because of this, it is critical that the effects of both air and noise pollution on pregnancy and fetal health are studied concurrently. Herein, we report for the first time, the effects of intermittent noise pollution during implantation (GD 5–6) with or without exposure to 0.4 ppm ozone on both maternal and fetal health outcomes in Long-Evans rats. Similar to our previous work [3], we demonstrated that exposure to 0.4 ppm ozone-alone resulted in significantly reduced male fetal weight and only a trend (nonsignificant) reduction of female fetal weights. However, contrary to our initial hypothesis, exposure to noise or to a combination of ozone and noise, elicited unanticipated uterine artery flow changes but did not result in reduced fetal weight.

In the current study, dams exposed to noise, ozone, or both during implantation receptivity failed to obtain the dynamic reduction in uterine arterial resistance between GD 15 and 21 as was observed in A + Q dams. While the failure to adequately reduce uterine arterial resistance occurred in the A + N group, fetal weight was only reduced in the males from O_3_ + Q dams. It may be significant that this group was also the only group to exhibit a significantly reduced RI at GD 15. As we have observed in our other studies [3, 4], peri-implantation ozone exposure (0.4 and 0.8 ppm) causes reduced uterine arterial resistance at GD 15. The lack of continued RI decrements from GD 15 to GD 21 in ozone-exposed dams may reflect a low RI in mid-pregnancy that reached its nadir and hence failed to decrease further during gestation. The clinical significance of this finding is not yet clear, but to our knowledge, this has not been described in IUGR pregnancies in humans nor animal models of preeclampsia and growth restriction (e.g., Dahl salt-sensitive rat and the stroke-prone spontaneously hypertensive rat).

In our investigations, we performed the first ultrasound at GD 15, which is when the blood compartment begins to expand in the rat [29]. A recent in silico model [30] demonstrated that high-velocity perfusion to the intervillous space reduces spiral arterial remodeling and induces trophoblast shedding from the placenta. We have not yet assessed spiral arterial flow in our model. However, if RI in the uterine artery is already somewhat reduced by GD 15 in dams exposed to ozone during implantation compared with air-exposed dams, this could result in localized injury to the spiral arteries and placenta, impeding their further remodeling. The lowered RI sustained in the O_3_ + Q dams may have also induced structural placental damage, in turn contributing to the reduction in fetal weight observed only in this group, and by contrast, not observed in dams exposed to intermittent noise (A + N). Moreover, the prematurely reduced perfusion within the uterine artery may trigger a compensatory mechanism to maintain resistance in the artery in mid-to-late gestation, which could also explain the lack of continued reduction in RI in the ozone-exposed groups. Current work is underway to better understand the dynamic shifts in remodeling of both the spiral and uterine arteries throughout the entirety of gestation, as this appears to be a critical process by which exposure to ozone may impair growth across fetal development.

Another consistent finding relative to our previous work is the reduced effect size in female fetuses from dams exposed to 0.4 ppm ozone, suggesting that females are less sensitive to growth restriction. This differs somewhat from what is observed following 0.8 ppm exposures to ozone on GD 5–6. As we have found, the higher exposure concentration appears to reduce male and female weight to the same extent overall [3, 4]. Sex differences in the risk of various adverse pregnancy outcomes have been reported. While a female predominance for IUGR diagnoses in neonates has been observed [31, 32], importantly, male newborns have an increased risk of neonatal complications and perinatal mortality [33]. Furthermore, many of the later-life health effects related to IUGR (e.g., cardiovascular diseases) are suggested to have a greater propensity to occur in males [34]. Purportedly, sex-specificity relates to differences in placental adaptation between males and females [34]. In the current study, we failed to find an effect of peri-implantation 0.4 ppm ozone exposure on many of the placental outcomes assessed herein, despite showing relatively consistent changes following a 0.8-ppm exposure [4]. However, we have yet to study the placenta histology to assess differences in the size of the placental layers or extent of vascularity. Placental structure is known to be somewhat different between males and females, and thus IUGR has been associated with differing pathologic changes depending on the sex of the affected neonate [33]. These observations further suggest that the structural and vascular formation of the placenta may be important in mediating the risk of growth restriction [35]. Further investigation is necessary to fully characterize any sexual dimorphism that may exist in the placenta following peri-implantation exposure to 0.4 ppm ozone, including but not limited to histological differences.

The main purpose of our study was to better understand the effects of noise, a common non-chemical stressor in communities with close proximity to major roads, on fetal growth outcomes in a Long-Evans rats. Exposure to traffic-related noise has long been associated with maladaptive cardiovascular effects [36, 37], attributable to HPA axis activation and elicitation of a stress response [10]. More recent epidemiological evidence has likewise reported positive associations with elevated noise pollution (> 85 dB) and adverse birth outcomes [13, 14, 38]. Such associations suggest the potential for excessive noise exposure to be an independent risk factor for IUGR. While our data showed that exposure to noise during implantation lead to impaired reduction in uterine arterial resistance between GD 15 and 21 and had some effect on placental antioxidant status, noise exposure at the levels and duration used herein did not impair fetal weight gain. By contrast, another group found that mice exposed to noise (100 dB) on GD 7 had reduced fetal weight, which was blunted if dams were anesthetized throughout the exposure [16]. Discrepancies between these studies may relate to a difference in noise sensitivity between species, the magnitude and duration of noise exposure, or gestational timing. Nonetheless, a recent World Health Organization meta-analysis found a low quality of evidence to an association between noise and adverse birth outcomes [18]. In the report, it was noted that air pollution was likely a confounding factor in many epidemiological studies, which was further confirmed by Smith et al. [19] and Nieuwenhuijsen et al. [39]. Hence, the lack of a direct or interactive effect of noise pollution on fetal weight in our current study is quite consistent with recent epidemiological findings.

Although having little effect on its own, noise when added to ozone exposure altered responses relative to ozone alone, including divergent impacts on fetal weight. The specific interactions that occurred between ozone and noise, which may have driven these responses are unclear. Future studies will be necessary to understand the temporal effects of noise on critical processes in pregnancy immediately following exposure. Importantly, dams that exhibited respiratory distress (i.e., Penh > 0.75), appeared to be at an increased risk for small male fetuses at GD 21, suggesting perhaps that the greater the ozone-induced injury, the greater the effect on fetal weight. As the addition of noise reduced the effect size of the ozone exposure on Penh (*g*_Hedges_ = 1.15 vs. 2.95), it may be possible that noise exposure may have dampened the acute respiratory impact of ozone and thereby hindering its effect on fetal development.

We have recently investigated the extent of systemic injury in dams immediately following exposure to ozone during implantation receptivity and note that a reduction in circulating inflammatory cytokines may have occurred [6]. In addition to activation of the HPA axis, noise exposure may also be immunomodulatory in susceptible models [40, 41]. Hence, it is plausible that exposure to noise may be interacting with the acute effects of ozone (e.g., injury and resultant inflammation), or even moderating the impacts of ozone by triggering opposing mechanisms. While further work is needed to better define such mechanisms, our findings clearly indicate that exposure to ozone during implantation has unique effects on pregnancy, which may be modifiable by other environmental stressors such as noise.

In summary, we report that exposure to 0.4 ppm ozone (× 4 h) during implantation receptivity, a susceptible window that may modify the risk of adverse pregnancy outcomes [5], produces replicable reductions in the weight of male fetuses at GD 21. Similar to our previous investigation [3], exposure to 0.4 ppm during implantation receptivity did not result in a significant reduction in weight of females fetuses, thus suggesting a sex-specificity in the consequences of ozone exposure during implantation. Lastly, we propose that both noise and ozone exposure during implantation receptivity have independent effects on pregnancy, with ozone having the strongest impact on fetal weight.

## Perspectives and significance

The lack of an additive effect of exposure to both noise and a prototypic air pollutant (ozone) on fetal weight in the current study closely mirrors recent epidemiological associations indicating that the elevated risk of low birth weights in communities near roadways is attributable to increased exposure to traffic-related air pollutants and not exposure to noise [18, 19, 39]. Further work is needed to determine if interactive relationships may exist with other air pollutants (e.g., particulate matter or nitrogen oxides) and/or with more prolonged exposures to noise during gestation.

Lastly, as our data indicate, female offspring appear less susceptible to the effect of peri-implantation 0.4 ppm ozone exposure on fetal weight. This finding may agree with the epidemiological reports of reduced neonatal complications in the female sex [33], which is likely attributable to improved placental adaptability in females that others have theorized [34]. Considerable research is needed to better understand the sexual dimorphism of the placenta and how this may contribute to adaptive mechanisms upon encountering these or other environmental stressors.

## Data Availability

The datasets generated and/or analyzed during the current study will be made available on https://catalog.data.gov/ following final publication of this manuscript.
